# A Rare Case Report and Literature Review of Anabolic-Androgenic Steroids (AAS)-Induced Acute Myocardial Infarction

**DOI:** 10.7759/cureus.8332

**Published:** 2020-05-28

**Authors:** Qian Zhang, Khine S Shan, Ahmad Raza, Neelima Manda, Travis Nace

**Affiliations:** 1 Internal Medicine, Abington Hospital-Jefferson Health, Abington, USA; 2 Internal Medicine, University of Maryland Medical Center, Baltimore, USA; 3 Library Science, Abington Hospital-Jefferson Health, Abington, USA

**Keywords:** mi, myocardial infarction, st-elevation myocardial infarction (stemi), anabolic steroid, steroid, inflammatory bowel disease, crohn's disease, ulcerative colitis

## Abstract

Anabolic-androgenic steroids (AAS) abuse is common in competitive athletes in order to enhance athletic performances. However, AAS abuse is often associated with deleterious side effects including but not limited to cardiovascular diseases, depression, hormonal abnormalities, and cancer. We present a case of a 31-year-old male with a history of Crohn’s disease on infliximab and chronic AAS use who had persistent retrosternal chest pain found to have an acute myocardial infarction (MI) without obvious cardiovascular risk factors.

## Introduction

Anabolic-androgenic steroids (AAS) are synthetic drugs manufactured to mimic the male sex hormone testosterone [1]. They are usually prescribed for medical conditions including but not limited to impotence in males, endometriosis, hypogonadism, and aplastic anemia [2]. It has also been used historically by athletes and bodybuilders to enhance performance as it can stimulate protein synthesis leading to an increase in muscle size and strength [3]. However, the use of AAS has become a serious global public health dilemma as it has been used widely by the general population to improve physical strength and appearance [1]. In 2013, the United States Centers for Disease Control and Prevention (CDC) reported that 3.2% of high school students had taken AAS without a doctor's prescription at least once in their lifetime [4]. In a meta-analysis of 187 studies, the overall global lifetime prevalence rate of AAS use was 3.3% and it is higher in men (6.4%) than in women (1.6%) [1]. Approximately one million people, predominantly males, had developed AAS use dependence [5]. AAS use is associated with an increase in cardiovascular diseases with the rising incidence of myocardial infarction (MI) in young patients with a history of anabolic steroid use. Unfortunately, the abuse of AAS has remained on the rise despite their well-known deleterious effects. Here, we present a case of an acute MI in a 31-year-old man who was found to have cyclic AAS use while being treated with infliximab for Crohn's disease.

## Case presentation

Our patient was a 31-year-old man who presented to a regional hospital with the chief complaint of substernal chest pain. His past medical history was significant for Crohn’s disease treated with infliximab every six weeks. He was in his normal state of health until he suddenly developed substernal chest pain associated with nausea and vomiting after he finished his routine workout in the morning. He reported similar episodes of chest pain along with shortness of breath on exertion over the past two years. He was referred to a cardiologist who opted to perform an exercise treadmill stress test with unremarkable results. He denied any family history of cardiovascular diseases. He regularly followed a gastroenterologist for his Crohn’s disease that was complicated by anal fistula. Unfortunately, he had been experiencing daily chronic diarrhea due to Crohn’s disease. He was also scheduled for an abdominal ultrasound for his chronically elevated liver enzymes in the upcoming months. Otherwise, he denied alcohol abuse, smoking history, or illicit medication use. He worked as a security guard and was also a heavy weight lifter. He used chronic anabolic steroids in three-to-four months cycles for approximately the past ten years.

**Figure 1:**
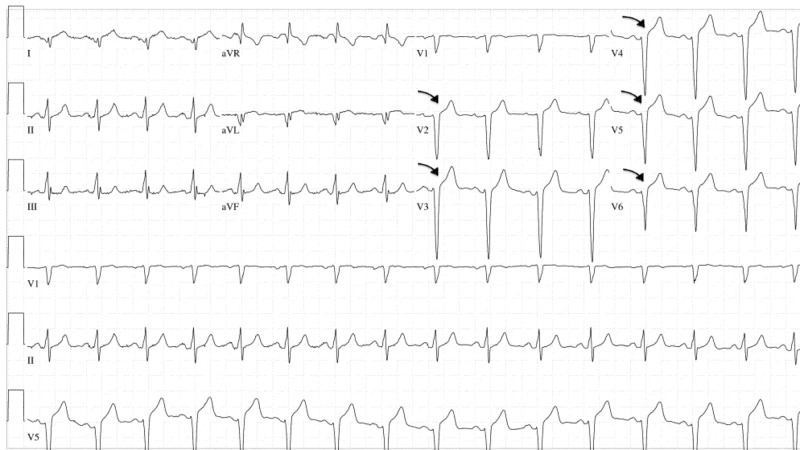
Electrocardiography (EKG) ST-segment elevation of the precordial leads of the EKG consistent with anterior myocardial infarction.

**Figure 2:**
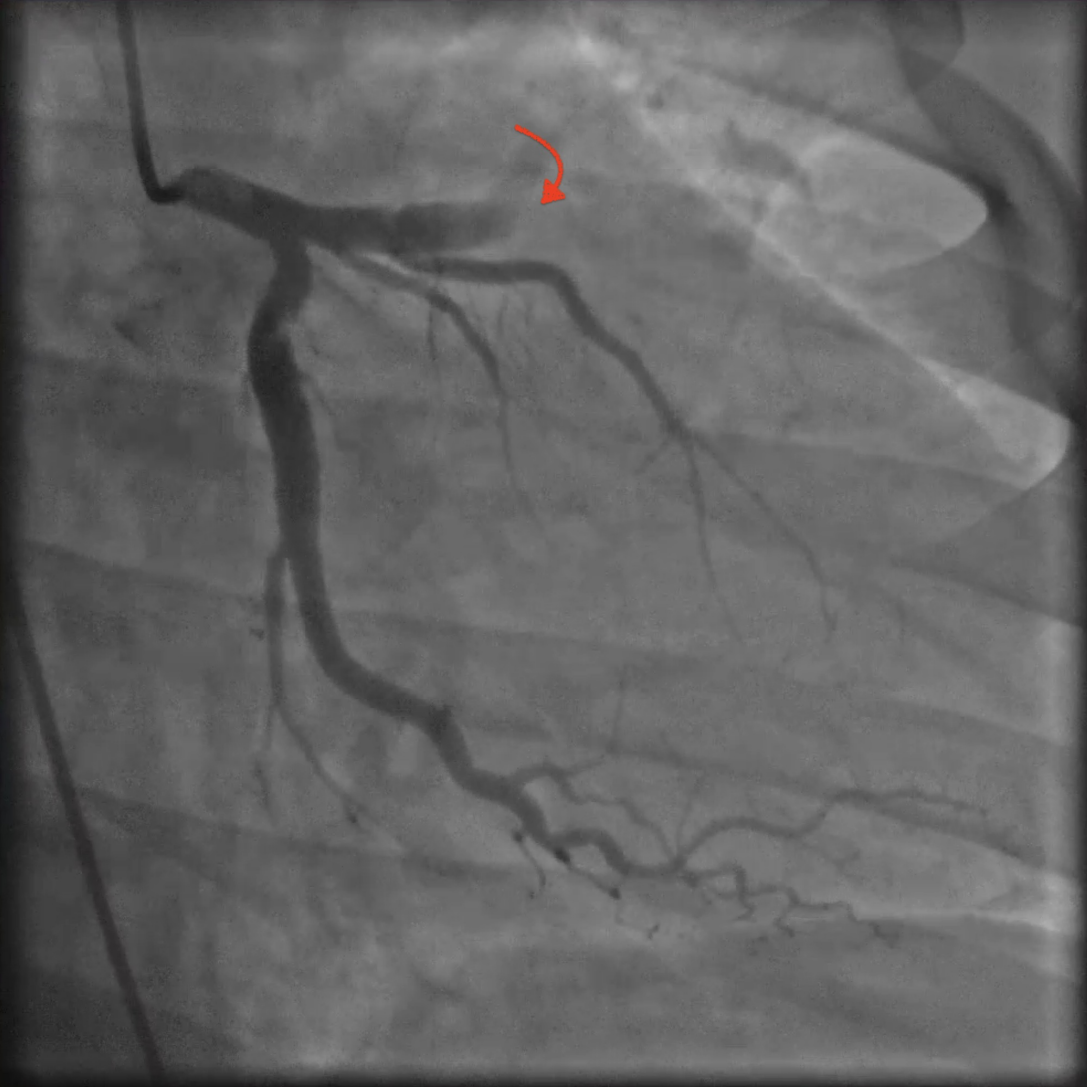
Coronary angiography Coronary angiography showed thrombotic occlusion of the left anterior descending coronary artery (LAD).

**Figure 3:**
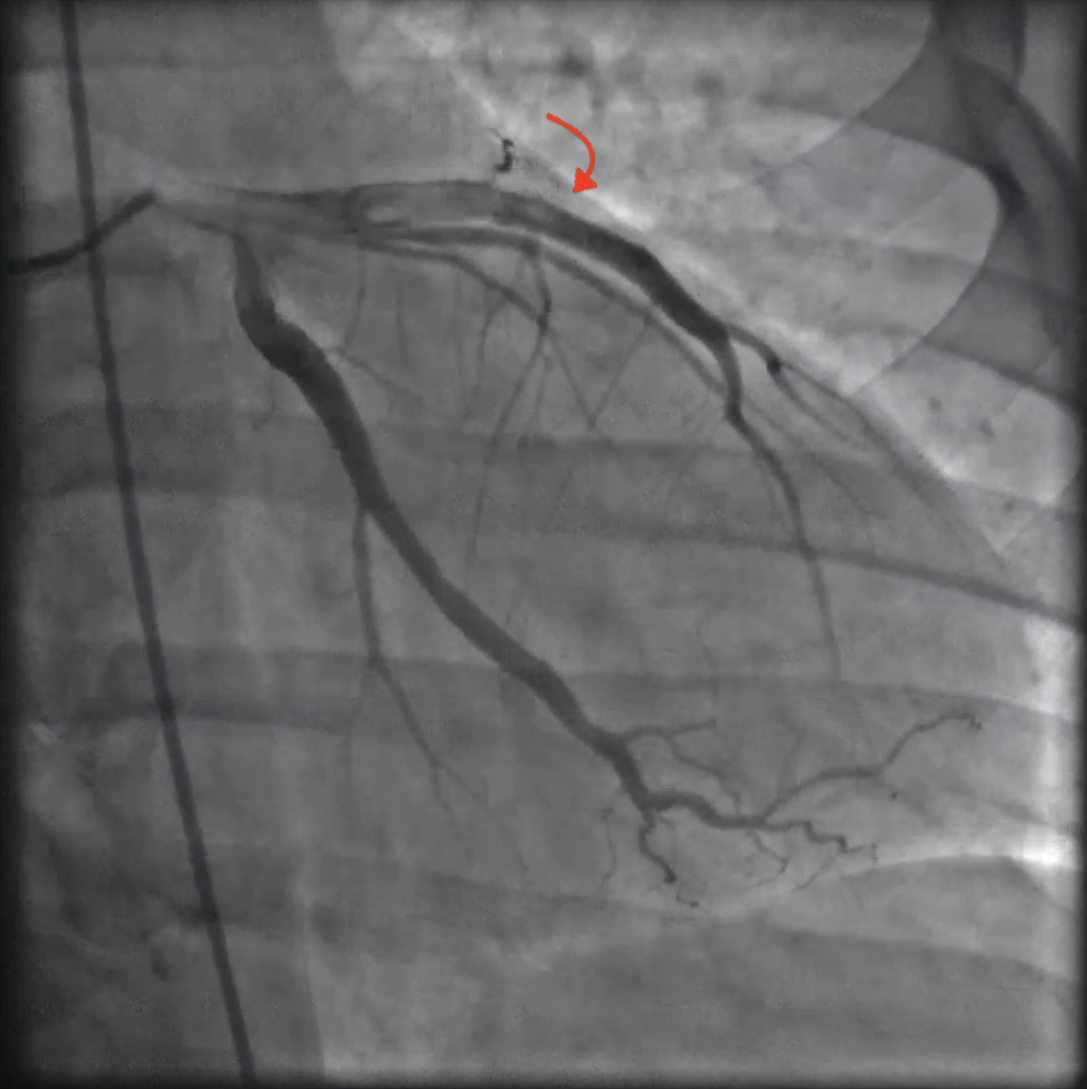
Coronary angiography Coronary angiography status post balloon angioplasty and bare-metal stent deployment showed revascularization of the left anterior descending coronary artery (LAD).

Electrocardiogram (EKG) revealed anterior wall ST-segment elevations along with an elevated cardiac troponin level (Figure 1). The patient was transferred to our hospital status post administering of aspirin 324 milligrams (mg) via helicopter transportation service in order to undergo emergent coronary angiography that revealed a normal left main coronary artery, but a thrombotic occlusion was found in the left anterior descending artery (LAD) (Figure 2). There was thrombolysis in myocardial infarction (TIMI) grade flow of 1-2 from collateral arteries of the distal right coronary artery to the septal cascade. The patient underwent balloon angioplasty associated with intracoronary thrombolysis of tissue plasminogen activator (tPA) 10 mg over 10 minutes. Insertion of a 4.5 x 3.8 millimeters of the bare-metal stent was deployed at high atmospheric pressure. The subsequent angiography demonstrated TIMI II/III flow to the anterior lateral wall with respective correlation found on the left ventriculogram that showed severe anterior lateral, anterior apical, inferior apical hypokinesis with an approximate ejection fraction (EF) of 30% (Figure 3). He remained to be hemodynamically stable throughout the procedure without significant arrhythmias except for a transient accelerated idioventricular rhythm (AIVR) episode. The patient was subsequently transferred to the cardiac care unit (CCU) status post the intervention for further management. He remained to be stable overnight.

On day two of hospitalization, he was hemodynamically stable with normal sinus rhythm while on telemetry monitoring. Laboratory results were unremarkable except for elevated troponin levels that peaked at 440 NG/ML, reactive leukocytosis of 18.0 K/UL without a clinical impression of infection along with elevated aspartate aminotransferase (AST) of 521 U/L and alanine aminotransferase (ALT) of 186 U/L with a normal level of alkaline phosphatase. He had a normal hemoglobin A1c of 5.4% and elevated low-density lipoprotein cholesterol (LDL) of 158 MG/DL despite a normal cholesterol level of 198 MG/DL. Echocardiogram showed a moderate to severely reduced left ventricular systolic function with an estimated EF of 25%-30%. He had no signs of acute congestive heart failure or arrhythmias. He remained to be stable overnight. 

**Figure 4:**
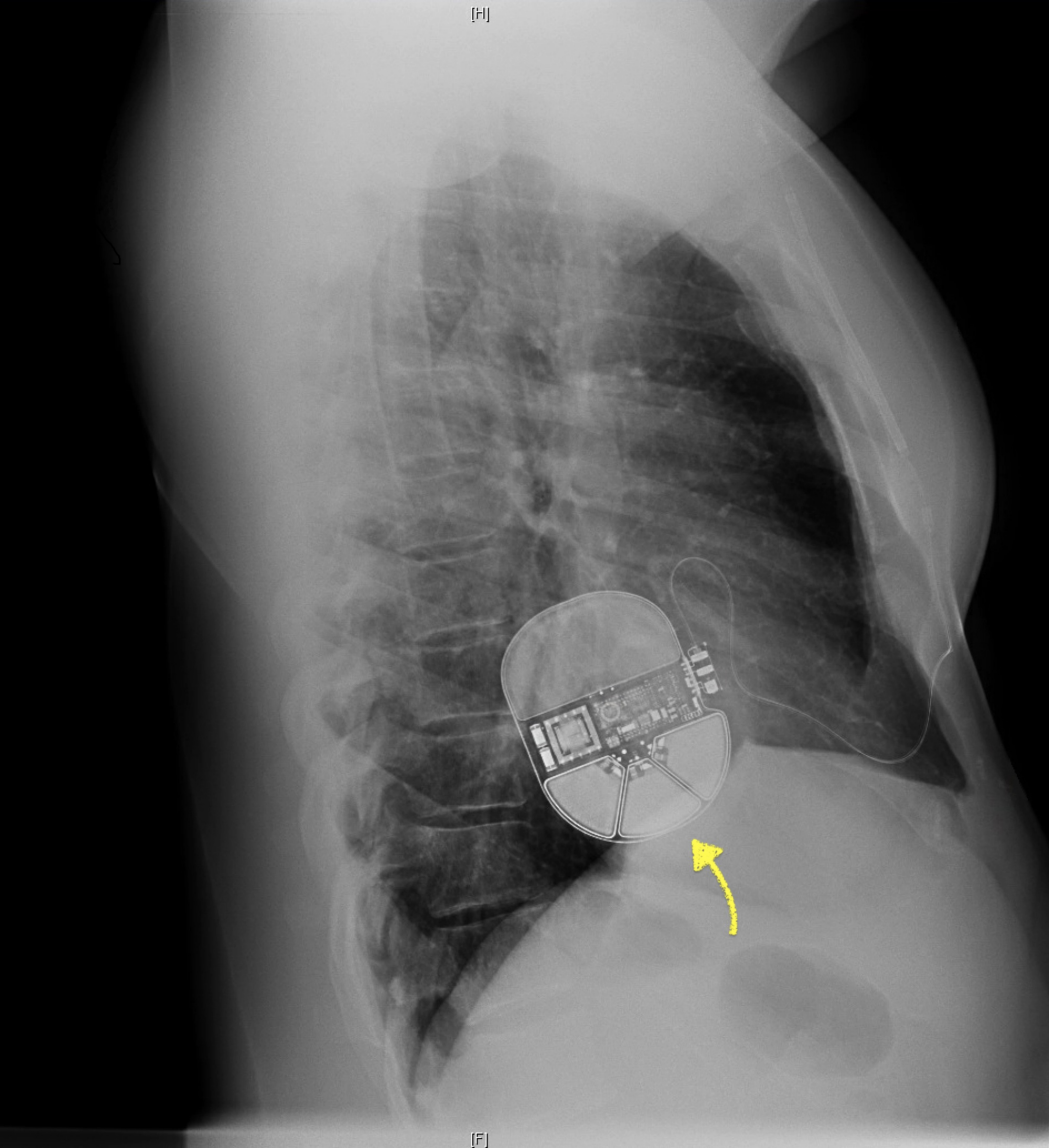
Automatic implantable cardioverter-defibrillator (AICD) Lateral view of chest X-ray showed intact left-sided AICD without evidence of active cardiopulmonary disease processes. H: head. F: foot.

On day three of hospitalization, he continued to remain hemodynamically stable without signs of arrhythmia or acute congestive heart failure. He was asymptomatic with negative reviews of the system. He was fitted for a wearable cardiac defibrillator to potentially reduce the risk of sudden cardiac arrest. He was subsequently downgraded to the telemetry floor from the CCU. He was discharged from the hospital and recommended against taking further anabolic steroids as it was believed to be the cause of his MI without obvious cardiovascular risk factors. He was advised to follow up with his primary cardiologist in the outpatient setting for the continuation of care. He eventually underwent an automatic implantable cardioverter-defibrillator (AICD) implantation three months later (Figure 4).

## Discussion

There are a total of less than 30 case reports of AAS associated MI in healthy young adults as of today [6]. Most of the reported cases were related to high doses of steroid intake (usually greater than five times as the recommended dose) in drug abusers and thus had a higher incidence of developing complications. Moreover, AAS poses various side effects that target the liver and cardiovascular system when the dose exceeds the capacity of our physiological levels. Potential side effects include but do not limit to dyslipidemia, hypertension, a decrease in cardiac output, hypercoagulability, testicular atrophy, gynecomastia, depression, aggression, increased risk of tendon tears and hepatic carcinoma [3,7]. These side effects may be reversible upon discontinuation of AAS. Furthermore, previous case reports described the association of AAS use with acute MI, sudden cardiac death, cardiac conduction abnormalities, cardiomyopathy, and cardiovascular thrombosis [5,6]. However, the frequency of cardiovascular events among patients taking AAS is likely underreported due to the patient’s preference in masking AAS abuse. 

The mechanism of actions between AAS and MI is currently not well understood despite the presence of previous case reports describing the association of MI in young adults with AAS use. One hypothesis revolves around atherosclerosis, thrombosis, direct endothelial injury, or vasospasm. AAS may cause accelerated atherosclerosis via their effects on lipid metabolism as LDL levels were found to be increased by 36% while high-density lipoprotein (HDL) levels were decreased by 52% in patients taking a high dose of AAS [8]. In addition, AAS can also enhance platelet aggregation likely via increased platelet production of thromboxane A2 (potent platelet aggregator) and/or decreased platelet production of prostaglandin 2 (inhibitor of platelet aggregation) [6,9]. Experimental studies have shown that animals treated with AAS had greater clot size and shorter vessel-occlusion time in response to thrombotic stimuli [6]. Furthermore, androgens may cause increased levels of procoagulant factors that lead to prothrombotic effects through coagulation/fibrinolytic cascade even though their effects on coagulation cascade and fibrinolytic pathway are currently not well understood [6,9]. Moreover, elevated homocysteine levels were found in a few cases of AAS-associated MI. Homocysteine has both atherosclerotic and thrombotic effects in healthy adults as it is toxic to endothelial cells by causing smooth muscle proliferation in the vessel walls and affecting the coagulation cascade [10]. AAS can also affect the absorption of Vitamin B 12 leading to hyperhomocysteinemia. AAS may also stimulate erythropoiesis which results in polycythemia which in turn increases blood viscosity leading to thrombosis [6]. Direct toxicity of AAS can also result in fibrosis and intimal hyperplasia of the intramural coronary arteries [11].

In patients with normal coronary arteries, the cause of MI may be due to coronary spasm as AAS can directly affect vascular endothelial cells resulting in vasospasm. Experimental studies showed that AAS can cause a decreased response to vasodilators due to inhibition of endothelial guanylate cyclase which leads to the reduction of nitric oxide-mediated relaxation [12]. Chronic AAS (Nandrolone) therapy was also found to cause decreased thoracic aorta relaxation due to decreased concentrations of arterial endothelial cyclic guanosine monophosphate [11]. 

Some cases described normal coronary arteries in AAS users with MI but others showed thrombotic occlusion of coronary arteries. The first report of MI was reported in a 22-year-old powerlifter who was found to have normal coronary arteries even though he had significant hypercholesterolemia and hyperactive platelet function [9]. However, another subsequent case described a young adult with a sudden death who was found to have thrombotic occlusion of the left main and left anterior descending coronary artery postmortem [9]. Hernandez et al. described a case of a 24-year-old man with a history of AAS abuse who suffered a cardiorespiratory arrest and was later found to have acute MI due to severe coronary atherosclerosis and superimposed acute occlusive thrombosis at the left main trunk and left anterior descendant [2]. Whereas another case described a 32-year-old man who had acute MI due to coronary spasm from AAS use as he was found to have normal coronary arteries on an angiogram [13]. A few other case reports described young men with AAS abuse who developed acute MI and was later found to have hyperhomocysteinemia [10,14]. Santos et al. mentioned a young man who was found to have an intraluminal thrombus likely due to a hypercoagulable state associated with AAS use [15].

AAS abuse has also been associated with both systolic and diastolic ventricular dysfunction. AAS abuse may cause direct myocardial injury causing increased collagen deposition, fibrosis and myocytolysis. It can cause ventricular hypertrophy via the upregulation of androgen receptors [2,6]. Our patient eventually received AICD placement due to his persistent systolic dysfunction in the three months follow up appointment. 

However, AAS may not be the only contributing factor for his acute MI despite his young age and lack of cardiovascular risk factors. Another factor that may be associated with his MI is his history of Crohn’s disease and Infliximab use. Even though there is currently no proven causal relationship between inflammatory bowel disease (IBD) and thromboembolic events, patients with IBD have a higher thromboembolic risk than the normal control population (8% vs 2%) with arterial thrombosis less common than venous thrombosis. In addition, vitamin B12 or folic acid deficiency, hyperhomocysteinemia, and increased lipoprotein associated with IBD may increase the risk of thrombosis. There were case reports describing IBD patients who were treated with Infliximab but had a higher risk of thrombosis with subsequent MI [16]. 

Our patient was a physically active young man without obvious cardiovascular risk factors along with a normal stress test two years ago. His acute MI was thought to be secondary to chronic AAS use as well as a possible contributing factor of Infliximab used for Crohn’s disease treatment

## Conclusions

It is important to raise awareness of the potential side effects of chronic AAS as it may lead to the development of MI. A detailed social history in the young patient population may be a game-changer when investigating the underlying causes of MI.
